# Silicon Carbide-Based Hydrogen Gas Sensors for High-Temperature Applications

**DOI:** 10.3390/s131013575

**Published:** 2013-10-09

**Authors:** Seongjeen Kim, Jehoon Choi, Minsoo Jung, Sungjae Joo, Sangchoel Kim

**Affiliations:** 1 Department of Computer Engineering, Kyungnam University, Changwon 631-701, Korea; E-Mails: cjh@keri.re.kr (J.C.); msjung@kyungnam.ac.kr (M.J.); 2 Creative Fundamental Research Division, Korea Electrotechnology Research Institute (KERI), Changwon 642-120, Korea; E-Mails: sj_joo@keri.re.kr (S.J.); sckim@keri.re.kr (S.K.)

**Keywords:** hydrogen sensor, high temperature, SiC, Ta_2_O_5_, MIS structure

## Abstract

We investigated SiC-based hydrogen gas sensors with metal-insulator-semiconductor (MIS) structure for high temperature process monitoring and leak detection applications in fields such as the automotive, chemical and petroleum industries. In this work, a thin tantalum oxide (Ta_2_O_5_) layer was exploited with the purpose of sensitivity improvement, because tantalum oxide has good stability at high temperature with high permeability for hydrogen gas. Silicon carbide (SiC) was used as a substrate for high-temperature applications. We fabricated Pd/Ta_2_O_5_/SiC-based hydrogen gas sensors, and the dependence of their I-V characteristics and capacitance response properties on hydrogen concentrations were analyzed in the temperature range from room temperature to 500 °C. According to the results, our sensor shows promising performance for hydrogen gas detection at high temperatures.

## 1. Introduction

There are many structures that can be employed for gas sensing applications, for example, metal oxides, conductive polymers, nano-structure and porous semiconductors [1–5] although conductive polymer-based sensors are not well suited for high temperature applications. Among them, metal-oxide semiconductors have been considered as typical gas sensing elements because of their good sensitivity and reliability. Most metal-oxide semiconductor gas sensors are based on the principle that when the sensor reacts with specific gas molecules, the surface of the sensor undergoes certain changes, which in turn result in some changes in the electrical properties of the sensor, such as its resistance or capacitance. These sensors usually operate at an elevated temperature for maximum performance.

Today, hydrogen has many important applications, such as its use in the processes of many industries, including chemicals, petroleum, food and semiconductors. Furthermore, the negative environmental impacts of burning fossil fuels, coupled with rising oil prices, have led to renewed interest in clean energy technologies, especially those involving hydrogen, but there are some obstacles related to safety that must be overcome to realize the potential of hydrogen as a fuel source. By nature, hydrogen is explosive, and its low mass and high diffusivity makes it difficult to store. If hydrogen flows into the air from a tank or valve, it will pose a hazard. Therefore, it is of great importance to detect hydrogen leakages at levels below the lower explosive limit of a 4% volume ratio of hydrogen to air. Hydrogen is also a major cause of corrosion. This happens when hydrogen atoms penetrate into steel and other metals and deteriorate the metals internally. This effect is especially pronounced at elevated temperature. Currently, hydrogen gas sensors are in wide demand as essential components in industries such as glass, chemical and petroleum industries that require storage tanks and refining processes, where the leakage of hydrogen is unavoidable. Besides, hydrogen sensors have been required for direct monitoring of the processes such as automotive exhaust parts, where temperatures may exceed 500 °C, but there are few of devices capable of operating at this high temperature. Hence, investigations on devices as well as materials and sensing structures capable of withstanding such conditions are needed.

Silicon has been the most widely used material as a substrate for semiconductor devices. However, due to its narrow bandgap, the maximum operating temperature of Si is limited to below 250 °C, which restricts its use in specific high-temperature environments. Alternatively, silicon carbide (SiC) has emerged as the leading candidate substrate for high-temperature operation. Its wide band gap, chemical inertness and stability have made it more ideal for high-temperature applications. As a result, SiC has been now in the forefront of wide bandgap semiconductor research such as high-power devices [6,7]. Generally wide-bandgap semiconductors allow also high-temperature operation up to 1,000 °C. This property of SiC allows hydrogen sensors [8–12] based on this material to be integrated with high-temperature electronic devices on the same chip. Moreover, it has excellent thermal conductivity (3–4.9 W/cmK), chemical inertness and radiation hardness. This unique quality offers the chance to eliminate or at least minimize the expensive bulky cooling systems that protect electronic devices from harsh environments.

Until now, metal-oxide hydrogen sensors [13–15] have been exploited mainly with the dielectric films of silicon oxide (SiO_2_), tin oxide (SnO_2_), and titanium oxide (TiO_2_), and they have usually been fabricated by resistive-type for high sensitivity and fast response time. In this work, a capacitive-type hydrogen sensor using thin tantalum oxide (Ta_2_O_5_) to replace these films as a dielectric material is introduced. For the improvement of sensitivity, the effective choice of dielectric material is important. Tantalum oxide [16] displays high permeability for hydrogen gas, high dielectric permittivity and low absorption for light. In the experiments described herein the tantalum oxide was formed by oxidizing tantalum (Ta) sputtered on SiC substrates by rapid thermal processing (RTP) in an atmosphere containing oxygen.

## 2. Experimental

### 2.1. Device Fabrication

To fabricate capacitive-type hydrogen sensors with MIS structure, we used 4H-SiC wafers as substrates. Electronic devices fabricated on 4H-SiC substrates have shown promise due to the greater availability and quality of reproducible single-crystal wafers, compared to other polytypes of SiC substrates. In addition, the wide band gap of 4H-SiC provides an advantage of dramatically reducing the number of electron-hole pairs formed from thermal activation across the band gap, which allows the high-temperature operation of electronic devices, including sensors.

**Figure 1.**
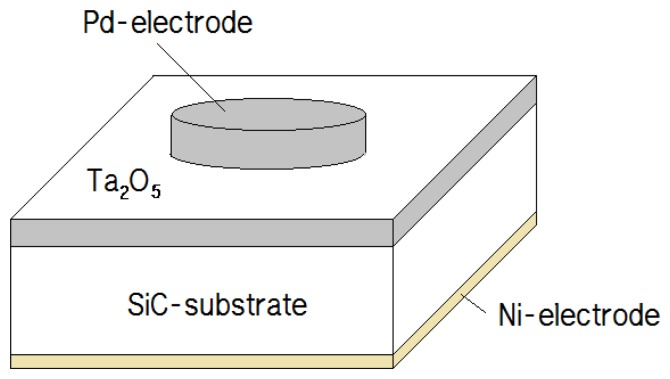
Schematic diagram of the hydrogen sensor.

After cleaning the samples, a tantalum (Ta) target was sputtered on 1 cm^2^ SiC substrates for 2 min with 300 W of power. The tantalum was oxidized by rapid thermal processing (RTP) at 500 °C for 3 min in an atmosphere containing oxygen. Under the controlled oxidation conditions, the thermally grown tantalum oxide was found to be very reproducible and very stable chemically. After the oxidation, Ni was deposited as a back-side electrode by sputtering for 20 min with 300 W of power, followed by RTP at 950 °C for 1 min to stabilize the Ni electrode. Next, palladium (Pd) was deposited on the Ta_2_O_5_ tin film with a shadow mask. The choice of catalytic metals (in this case Pd) depends on the nature of the gas to be detected. In this work, a Pd metal electrode is used as a catalyst for the chemisorption of hydrogen on the surface, because it has been known that Pd has very high solubility for hydrogen, and that the diffusion of hydrogen through Pd is very fast. The adsorption of hydrogen in a metal depends on the temperature and hydrogen concentration. Figure 1 shows a diagram of our hydrogen sensor, which is fabricated into an SiC-based MIS capacitor with a Ta_2_O_5_ dielectric layer and a Pd upper-electrode with a diameter of 5 mm, and an Ni lower-electrode with an area of 1 cm^2^.

### 2.2. Measurements

**Figure 2.**
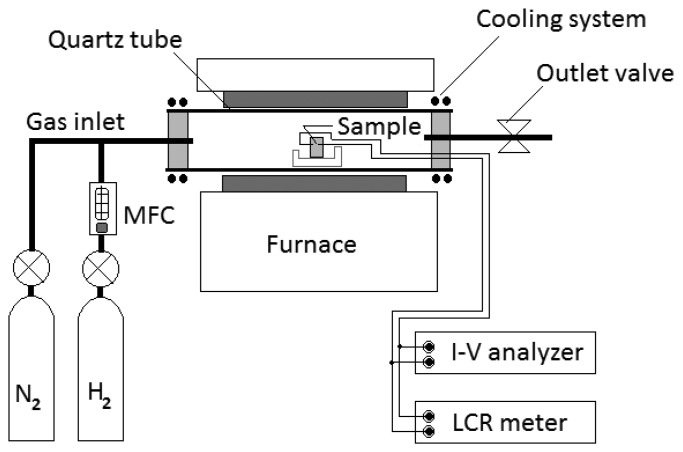
A set of equipment for testing hydrogen sensors.

Electrical measurements for sensors were carried out with a semiconductor device analyzer and an LCR meter. The sensors were measured in a chamber in which the temperature can be controlled from 150 to 700 °C. The chamber was composed of a quartz tube of 75 mm diameter with a cooling system. Hydrogen gas was injected through a hydrogen MFC (mass flow controller). Hydrogen concentrations in a chamber were varied up to 2,000 ppm in temperatures ranging from room temperature to 500 °C. The sensors were flushed with clean nitrogen gas before exposure to hydrogen gas. Figure 2 shows the equipment set-up for testing our hydrogen gas sensors.

## 3. Results and Discussion

**Figure 3.**
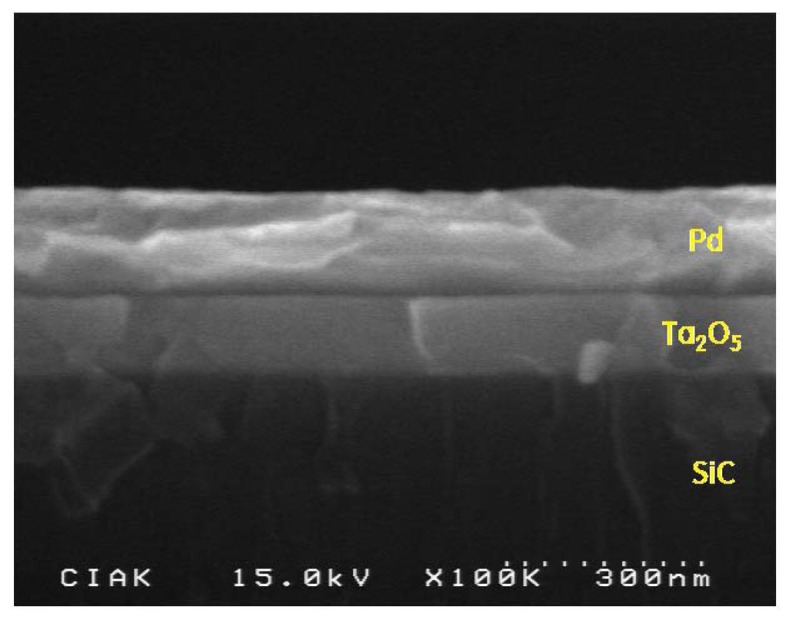
SEM image of the sensor.

Our hydrogen sensor was fabricated into an SiC-based MIS capacitor composed of a thin Ta_2_O_5_ dielectric layer of 120 nm thickness and a Pd electrode of 160 nm thickness, as shown in the SEM image in Figure 3.

A MIS-capacitor sensor is a simple device with a catalytic metal electrode that is very sensitive to low gas concentrations, and it has a compact structure that can be economically mass-produced via microelectronic fabrication techniques. Such devices are attractive due to their chemical specificity and high sensitivity to specific gases. They can operate even at elevated temperature with high degrees of sensitivity and selectivity.

The thickness of the sandwiching dielectric layer in devices determines whether the behavior of a device is diode-type or capacitor-type. For a capacitor-type response, the dielectric layer must be a good insulator, and capable of providing a huge tunneling barrier for the carriers. Generally, devices that have a dielectric layer of 100 nm thickness may be grouped as either MIS capacitors or Schottky diodes according to their working principle and applied voltage.

**Figure 4.**
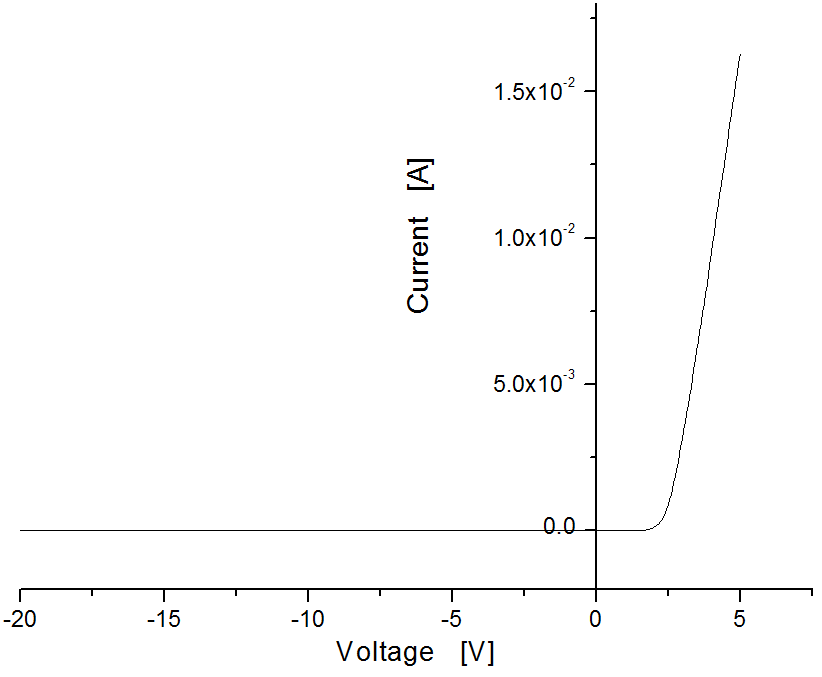
I-V curve observed at room temperature.

Figure 4 shows the current-voltage (I-V) curve measured in the sensor with MIS structure. Under applied voltages ranging from −20 V to 5 V at room temperature without hydrogen injection, the sensor showed rectifying characteristics like a Schottky barrier, with a turn-on voltage of around 2 V.

**Figure 5.**
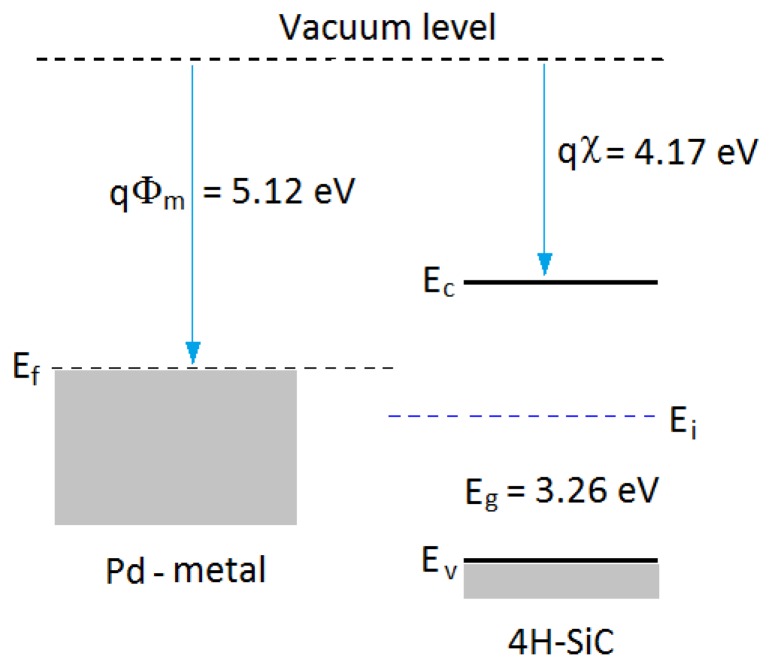
Energy band diagram for isolated Pd-metal and n-type SiC-semiconductor structure.

In general, a Schottky barrier is a metal-semiconductor contact with a large barrier height. The potential barrier between the metal and the semiconductor can be identified on an energy band diagram. The energy band diagram of Pd-metal and SiC-semiconductor under the same vacuum level is given as shown in Figure 5. The barrier height (Φ_B_) from the metal toward semiconductor is defined as the potential difference between the Fermi energy (E_f_) of the metal and the band edge where the electron majority carriers reside. For n-type semiconductors, the barrier height is obtained from:

(1) $$\Phi_{B}=\Phi_{\text{m}}-\chi$$

where Φ_m_ is the work function of the metal and χ is the electron affinity of the semiconductor. The potential barrier (V_bi_) from the semiconductor toward metal is defined as the potential difference between the Fermi energies of metal and semiconductor. In the case of Pd-SiC contact, Φ_B_ is much larger than V_bi_, so the sensor shows rectifying characteristics in that only leakage current exists in the reverse bias to −20V.

In our MIS capacitor, the total capacitance is given by the series addition of the oxide and semiconductor capacitance, C_OX_ and C_S_, respectively:

(2) $${\text{C}}_{\text{TOTAL}}=\frac{C_{ox}C_{s}}{C_{ox}+C_{s}}$$

**Figure 6.**
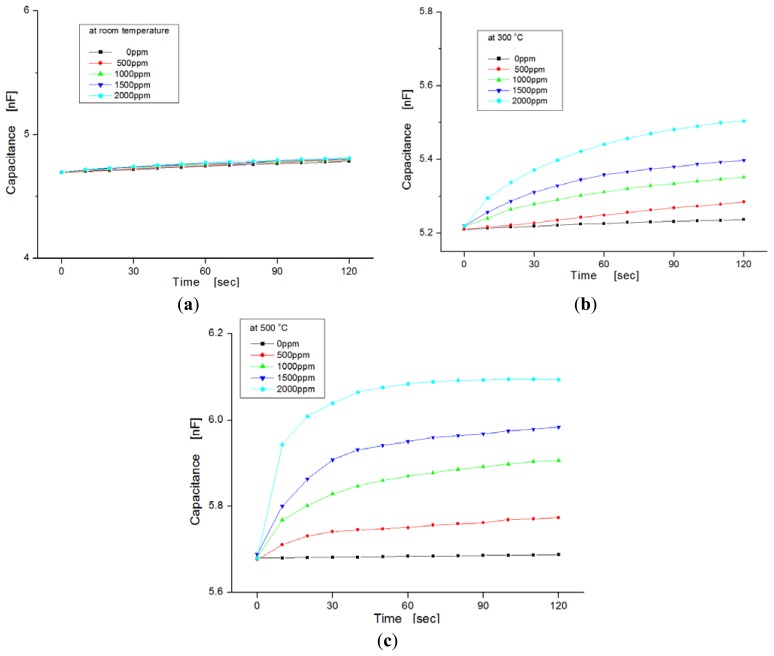
Dependence of capacitance on hydrogen concentrations as a function of temperature.

Figure 6 shows the variation of capacitance for exposure to different hydrogen concentrations as a function of temperature ranging from room temperature to 500 °C. In this experiment, hydrogen concentrations were varied from zero to 2,000 ppm in steps of 500 ppm. We observed the dependence of the capacitance on injected hydrogen concentrations. At room temperature the capacitances in the samples were nearly invariable, regardless of hydrogen concentrations, as shown in Figure 6a, but at temperatures of 300 °C and 500 °C, the capacitance changed more for the hydrogen concentrations with increases of temperature, as shown in Figure 6b,c, respectively. At a temperature of 500 °C, the slope of the variation increased more than at room temperature. Generally, the response time of the sensor was reduced as the variation in capacitance was saturated quickly, and the sensitivity was improved at elevated temperatures because the diffusion of hydrogen molecules to the dielectric layer is fast, and any sticking of water molecules to the metal surface is prevented.

**Figure 7.**
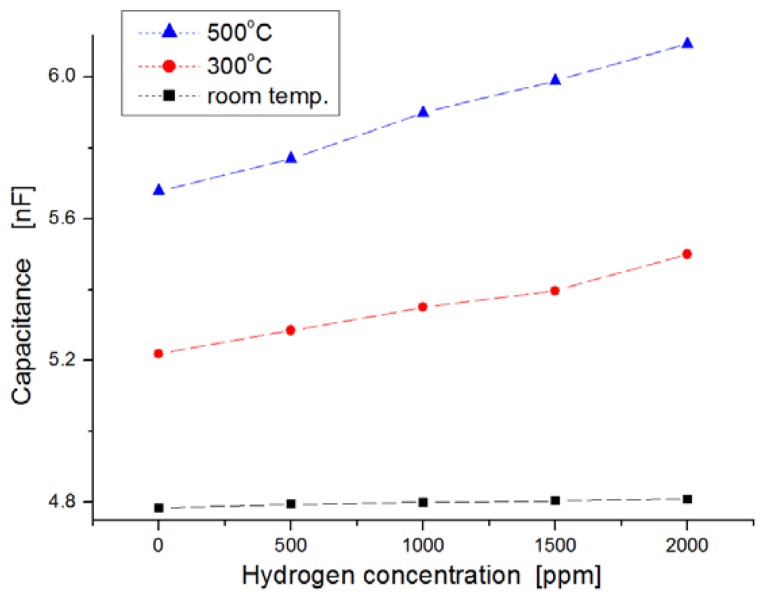
Dependence of capacitance on hydrogen concentrations as a function of temperature.

For harsh-environment applications, the operating temperature of sensors should be at least higher than 250 °C. Figure 7 shows the response behavior after a 120-s lapse in exposure to hydrogen gas. The variation of the capacitance in the sensor was observed extensively at the measurement temperature of 500 °C, while the variation in the sensor was nearly invariable at room temperature when the sensors were exposed to hydrogen gas molecules. At 500 °C, the average value of ΔC/Co was observed to be about 4 percent per 1,000 ppm hydrogen concentration (where Co indicates the value in capacitance at the state of zero hydrogen concentration, and ΔC is the variation in capacitance). Quantitatively, the capacitance increased from 5.6 nF to 6.1 nF when the hydrogen concentration increased from zero to 2,000 ppm at 500 °C.

## 4. Conclusions

There has been substantial study on the application of different dielectric layers in fabricating gas sensors, as sensing of different gases can be controlled by appropriate selection of the oxide layer. In this work, a tantalum oxide (Ta_2_O_5_) layer with high permeability for hydrogen gas was investigated to detect hydrogen gas in the atmosphere, and a SiC substrate was used for high-temperature applications at more than 300 °C. We fabricated Pd/Ta_2_O_5_/SiC-based MIS devices, and measured both I-V curves and the variation of capacitance for exposure to different hydrogen concentrations as a function of temperature ranging from room temperature to 500 °C. In the I-V measurement, the sensor showed rectifying properties as expected. In the capacitance measurement, the maximum variation of capacitance was observed at 500 °C, where the average capacitance variation rate was observed to be about 4 percent per 1,000 ppm hydrogen concentration. In conclusion, our sensor exploiting a Ta_2_O_5_ dielectric layer showed possibilities with regard to use in hydrogen gas sensors for high-temperature applications.
